# Continuous low-intensity predation by owls (*Strix aluco*) on bats (*Nyctalus lasiopterus*) in Spain and the potential effect on bat colony stability

**DOI:** 10.1098/rsos.230309

**Published:** 2023-08-16

**Authors:** Detlev H. Kelm, Manuel Langheld, Jesús Nogueras, Ana G. Popa-Lisseanu, Carlos Ibáñez

**Affiliations:** Estación Biológica de Doñana (CSIC), Seville, Spain

**Keywords:** owl predation, anti-predator behaviour, roost-switching, roost fidelity, bat roosts, bat mortality

## Abstract

Owls prey on bats, but information on owl predation is scarce, its impact on bat mortality is unclear, and reports on behavioural responses, including roost-switching and fission–fusion behaviour, are equivocal. To study the link between owl predation and anti-predator behaviour in bats, we evaluated seven months of video recordings at roosts and the behaviour of 51 passive integrated transponder (PIT)-tagged bats and bats without tags in a geographically isolated colony of greater noctule bats (*Nyctalus lasiopterus*) in Spain. We found the tawny owl *Strix aluco* to almost continuously hunt *N. lasiopterus*, from perches and on the wing, well after the bats emerged at dusk and when they returned to their roosts. We recorded 39 unsuccessful and three successful attacks. Nonetheless, we found no evidence that owl predation modifies bat behaviour. While the bats constituted only a very small proportion of the owls' diet, owl predation accounted for an estimated 30–40% of bat mortality, which may have a significant impact on small, local or isolated bat populations, in particular, and thereby shape regional bat distributions. We hypothesize that low roost availability may also affect the bats’ potential response to predation, which could lead to natural predation having an excessive impact on bat populations.

## 1. Introduction

The predominantly nocturnal lifestyle of bats has been assumed to be a consequence of diurnal food competition or hyperthermia during daylight flight [1]. Another principal hypothesis is that nocturnality in bats results from high levels of predation during daylight [1]. Nevertheless, many predators have been found to prey on bats, including birds, mammals and reptiles [2–4]. Of these, only a few predators specialize to some degree on bats, including the palaeotropical bat hawk (*Macheiramphus alcinus*) and the neotropical bat falcon (*Falco rufigularis*), which hunt bats on the wing at dusk [5,6], and probably also some tropical owl species, like the black-and-white owl, *Ciccaba nigrolineata* [7], which appear capable of efficiently preying on active, flying bats in the night [8]. In the temperate zone, owls seem to prey frequently on bats [9]. Although many temperate owl species prey on bats, bats generally constitute only a minor proportion of their prey. In a literature review on the diets of 10 European owl species, Sieradski & Mikkola [9] estimated the average frequency of bats in the diet of owls to be below 0.2%. Exceptionally, the proportion of bats in the diet of owls can be much higher, when owl individuals specialize on bats. Individuals of three owl species in Europe have been found, at least temporarily or perhaps in exceptional situations, to include higher proportions of bats into their diet (up to 27% in barn owls *Tyto alba* [10]; up to 29% in long-eared owls *Asio otus* [11]; over 30% in tawny owls *Strix aluco* [9]). Despite the generally low proportion of bats in the owls' diets in the temperate zone, the impact of owl predation on bats may be ecologically significant, because of the bats’ life-history traits, including their low fecundity [12]. Speakman [13] estimated 11% annual bat mortality in Britain due to bird predation. In most cases, however, the ecological relevance of avian predation on bats cannot be fully assessed, because of the difficulty of collecting suitable data in the dark of night [14]. Most observations of avian predation on bats have been made during the bats' emergence from their roosts at dusk, or at large cave roosts, while behavioural observations of owl attacks on bats during the night are mostly lacking (however, see [15,16]).

Apart from the nocturnal lifestyle, the timing and clustering of bats’ emergence from roosts have also been suggested as strategies to avoid predation [17]. However, direct evidence of the effects of predation on emergence is equivocal [14]. Some studies have found predator presence at roosts to affect the temporal clustering of bats exiting their roost [18], while others did not show any clear influence of predators on roost departure times [19].

Roost-switching could also be an effective anti-predation strategy in tree-roosting bats, particularly if predators tend to revisit sites where predation success is high [20,21]. Indeed, frequent roost-switching, sometimes of entire roosting groups, is common in many bat species, but its triggers and drivers are poorly understood. Some studies have found thermal conditions and roost parasite infestation to trigger roost-switching in some species [22,23], whereas for others roost-switching has been hypothesized to promote social cohesion within the colony and to contribute to the transfer of information on roosts [24–26]. In some bat species, colonies split into subgroups that are distributed over several diurnal roosts, between which the colony members switch regularly, while colony membership stays consistent. This behaviour has been described as fission–fusion [24,26,27], and may depend on individual roosting strategies, as the effects of environmental factors or of the bats' physiological status on behaviour may vary between roost group members. Nevertheless, any type of anti-predator behaviour requires that the bats assess the predation risk either by directly observing predators or via information transfer between bats. In this regard, there is little information on how bats perceive predation and whether there is information transfer on predation among bats that triggers anti-predator behaviours, such as roost-switching.

The greater noctule, *Nyctalus lasiopterus*, is the largest bat on the European continent. This partly carnivorous species feeds on insects, and seasonally includes small migrating birds in its diet [28,29]. During reproduction, female *N. lasiopterus* aggregate in roosting colonies, i.e. social groups, in hollow trees and bat boxes. The members of roosting groups show fission–fusion behaviour, and switch between roosts and subgroups while maintaining the social cohesion of the colony [26]. However, the factors that trigger roost-switching and the level of information transfer between individuals are not clear.

The aims of this study were to (i) describe owl predation on *N. lasiopterus* at their roosts, (ii) assess whether owl presence and predation affect roost-switching or the timing of roost-emergence, and (iii) assess potential effects of owl predation on the size and stability of a colony of *N. lasiopterus*. We hypothesized that owl predation near roosts can trigger roost-switching, and thereby contribute to fission–fusion behaviour in this bat species. The coordinated abandonment of roosts by most or all individuals would also indicate information transfer between individuals on the predation event, especially in situations in which it is unlikely that all roost occupants directly observed the predation event.

## 2. Methods

### 2.1. Fieldwork

The study was carried out in the Doñana Biological Reserve (CSIC) in the Doñana National Park in the Province of Huelva (Andalusia), in southern Spain between 1 January and 18 July 2016.

**Figure 1.**
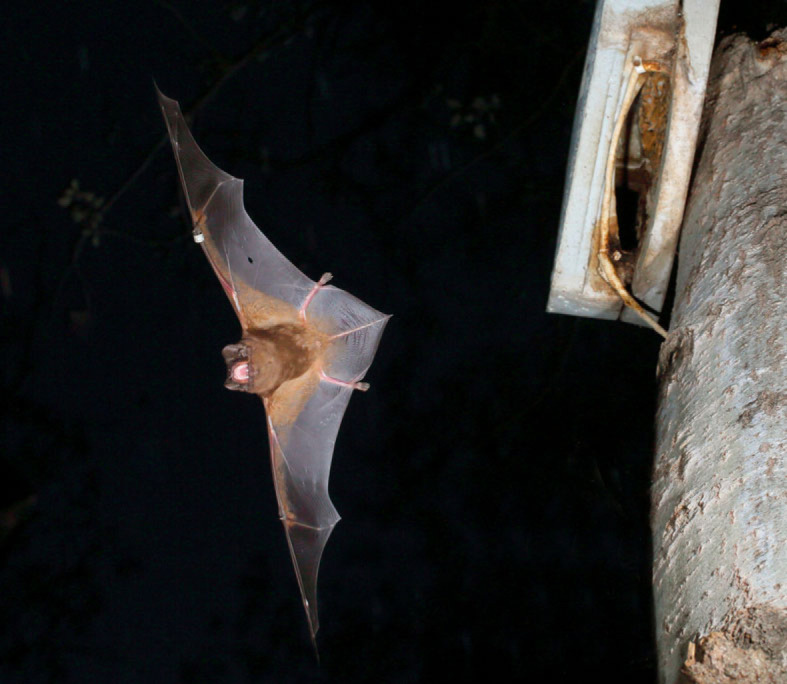
*Nyctalus lasiopterus* at a study roost (Schwegler 1FQ). The white cable around the roost entrance is the PIT tag reader antenna. Photo: Jens Rydell.

**Figure 2.**
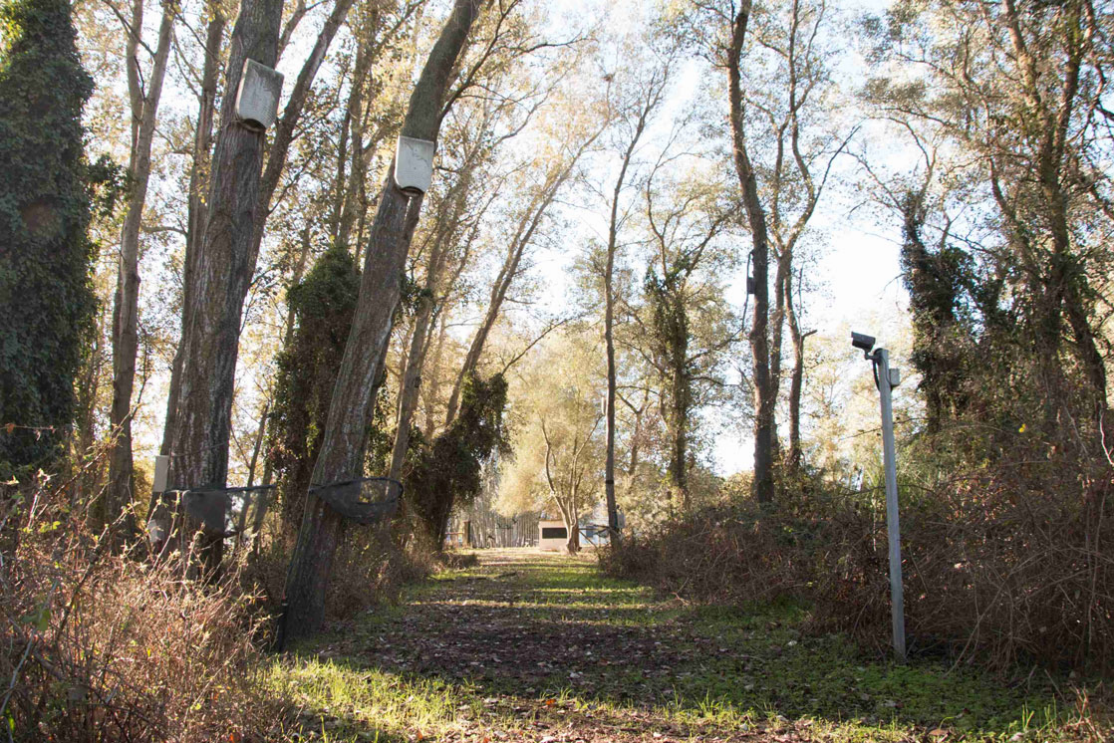
Artificial bat roosts and video camera. Photo: Elena Tena.

Previous observations and passive integrated transponder (PIT) tagging of bats showed that the study colony of *N. lasiopterus* included *ca* 50–60 individuals during the reproductive period from April to July. During the rest of the year, particularly between October and March, the colony was smaller, as most of the bats were at their wintering sites. As winters are mild in the study area (only 6 days had a minimum temperature less than 2°C, or a maximum temperature less than 15°C during the study period), the bats are mostly active throughout the year. Between 2002 and 2016, 260 bats in the study area have been individually marked with 5.2 mm aluminium alloy rings (Porzana, Ltd, Icklesham, UK) and subcutaneous microtransponders (ID-100A, Trovan EID Ibérica, Spain), and their biometric data has been recorded. In 2016, bats were only tagged after the study to prevent bat capture from biasing the behavioural data. Automatic PIT tag readers (LID650, Dorset Group, The Netherlands) at roost entrances allowed roost use by bat individuals to be continuously registered. The reader antenna was a ring around the roost entrance that registered bats entering and leaving the roost, or those remaining within *ca* 5 cm of the antenna (figure 1). During the study, the colony used 11 artificial roosts (seven Schwegler 1FQ (width 35 cm, height 60 cm, depth 9 cm, all of grey colour); one Schwegler 2FN (∅ 16 cm, black and grey); three custom-made cork roosts of similar size and natural colour) and a single natural roost in the hollow trunk of a eucalyptus tree. The artificial roosts were fixed to trees at a height of *ca* 4.5–6 m above ground within a forest patch of poplar (*Populus alba*) and remnant old eucalypts (*Eucalyptus globulus*) of *ca* 2 ha surrounded by shrubland and floodplain (36°59′28.5" N, 6°26′36.5" W). A gravel road partly encircled the forest patch and a footpath crossed it. The roosts were usually not more than 20 m away from a path (figure 2). Previous studies have found this colony to be partially connected to other nearby colonies [30]. The closest known breeding colonies of *N. lasiopterus* were in the cities of Jerez de la Frontera and Seville at distances of 42 and 59 km, respectively. Some individuals have been found to use roosts at both sites [30,31]. Some of the studied roosts have temporarily also been used by *Pipistrellus pygmaeus,* often with more than 50 individuals per roost, but we never observed both species roosting simultaneously.

At the seven most frequently used roosts, we installed motion-triggered infrared cameras (CDN-6022E, Golmar Sistemas, Spain) on posts of *ca* 2 m height at *ca* 5–15 m distances from the roost entrance and its surroundings. The analogue video signal was processed by an Axis Q7424 Video Encorder (Axis Communications, Sweden) and stored online or on local memory. When triggered, video recordings started 3 s before the event, and they continued until the end of the detected movement. To standardize the camera recordings at different roosts, we centred a similar-sized detection field on the roost via the software and calibrated the sensitivity of the motion trigger and the camera aperture with the bats entering and leaving the roost. All recordings included a time stamp, which was standardized to solar time (UTC), and we collected video data during 197 nights between January and July. No observer was present during the recordings. At two sites, two and three roosts, respectively, were less than 5 m apart. As video recordings of bats and predators in the vicinity of these roost sites could not be clearly assigned to a specific roost, data for all roosts at each of the two sites were lumped for statistical evaluation to avoid pseudo-replication. The moon phase was included as a decimal number in the dataset (0 = new moon, 1 = full moon).

### 2.2. Data analysis

We assessed the number of bats at each roost per day by analysing the video recordings to count all individuals emerging from the roosts at dusk, including bats without PIT tags. We defined the sum of all bats leaving the observed roosts as the minimum daily colony size. To assess seasonal effects, e.g. behavioural differences arising from seasonal variation in physiological status and reproduction, we divided the data into four time periods (seasons) taking into account the bats’ annual reproductive cycle: 1. ‘winter’, from January to mid-March, with a reduced number of bats and limited bat activity at the study site, 2. ‘spring’ until mid-April when bats return from their wintering areas and form the breeding colony, 3. the season of ‘pregnancy’ of the females until mid-May, 4. ‘lactation’ until the end of July when the offspring are weaned. By ‘pregnancy’ we refer to evident pregnancy (i.e. with a palpable fetus), because gestation lasts for more than a month and most females are already pregnant in the first half of April.

We ranked the observed owl behavioural events into six classes based on their assumed degree of disturbance at the bat roost (mild to severe): 1. passing the field of view, 2. perching in the field of view, 3. perching on the bat roost, 4. attack flight directed at the bat roost with the owl making contact with the roost without any bat visible (e.g. talons reaching into the roost), 5. failed bat capture attempt by an owl, 6. successful capture. Two observations were defined as separate events if they were more than 10 min apart, and observations of perching owls were recorded as a single event unless the owl left its perch and did not return within 10 min.

To model roost-switching, we defined two binary response variables, one at the roost level and a second at the individual level of a bat: 1. for each roost and day we assessed if the number of bats in the roost decreased compared with the previous day (variable ‘group size’), 2. for each day we assessed for each PIT-tagged individual if it had left the roost within the two days following an owl event (variable ‘gone’). We assumed that bats returning after an owl event may not always switch to an alternative roost during the same night. This may be particularly the case for lactating females, which have to return to the roost to nurse their young. Once inside the roost, they may decide to stay for the rest of the night before switching roosts the next night [32,33]. Female bats can carry their young to new roosts until these can fly [34,35]. To test for the effect of owl presence and predation on roost-switching by the bats, we defined two descriptive variables for owl predation: 1. the binary variable ‘owl presence’, i.e. an observation of any of the behavioural event categories 1–6, and 2. the variable ‘predation intensity’. We awarded one point for each observation of an owl event of class 1, two points for events of class 2 and 3, three points for event classes 4 and 5, and four points for events class 6, assuming a gradual increase in disturbance by owls at bat roosts. The variable ‘predation intensity’ is the sum of all points per roost and night.

We fitted Bayesian linear mixed-effects models (GLMM) using the ‘brms’ package v. 2.17.0 [36] in R (v. 4.1.3) with the default flat and weakly informative priors on the fixed and random effects respectively, and a Bernoulli error structure (4000 iterations, four chains, no thinning) [37]. We modelled the response variable ‘group size’ as a function of the independent variables ‘predation intensity’, ‘owl presence’ and ‘season’ and the interaction between the latter two factors. To account for repeated measures, roost identity was included as a random term. We modelled roost-switching at the level of the individual bat with a similar model, but with the incidence of bat individuals in a roost after an owl event as the dependent variable (response variable ‘gone’), and predation intensity, the number of bats in the roost, season and the sex of the observed bat individual as fixed effects, and the interaction of the latter two factors. We included roost identity and the identity of the bat individual as random effects on the intercept. We tested the fit of all models by applying posterior predictive checks, testing distribution, dispersion and outliers, and plotting the residuals against the predicted values with the DHARMa package in R (v. 0.4.5) [38].

We recorded three owl species perching at the roosts (tawny owl, *Strix aluco*; little owl, *Athene noctua*; barn owl, *Tyto alba*), two of which (*S. aluco*, *A. noctua*) were observed preying on bats, but *A. noctua* was observed to prey on pipistrelle bats (*P. pygmaeus*) exclusively. As the occurrence of different owl and bat species may confound the interpretation of *N. lasiopterus* behaviour, we assembled three different datasets. The first dataset (a) included all owl observations independent of owl or bat prey species. In the second dataset (b), we only considered interactions between *S. aluco* and *N. lasiopterus* in defining owl presence and calculating the variable ‘predation intensity’. The third dataset (c) was similar to (b), but we excluded all days of observations with *A. noctua* (*n* = 13). We ran models at the roost level (dependent variable ‘group size’) and at the level of the individual bat (dependent variable ‘gone’), resulting in six models ('group size a’, ‘group size b’, ‘group size c’, and ‘gone a’, ‘gone b’, ‘gone c').

To assess differences in *S. aluco* presence between roosts and over time, in another GLMM we modelled ‘owl presence’ (*S. aluco* presence) as a function of the number of bats in the roost, season and moon phase (model ‘owl presence’). In this model we included the roost identity as a random effect on the intercept.

To assess the effect of owl predation on the timing of emergence of individual *N. lasiopterus*, we first plotted the density function of bat emergence time after sunset, which took the form of a leptokurtic curve with a positive skew and long right tail (electronic supplementary material, figure S1). We then calculated the 75% quantile of the data, as 25% of emergences fell within the tail of the distribution. We defined a binary response variable ‘late emergence’ that indicated whether an emergence time fell within the tail of the curve. We included in a GLMM as independent variables ‘predation intensity’, ‘owl presence’, the number of bats in the roost, season and sex, as described before, and roost and bat identity as random terms (model ‘emergence’). The dataset included all observation days, and only interactions between *S. aluco* and *N. lasiopterus* were used to define owl presence and the variable ‘predation intensity’ (dataset b).

We tested for seasonal differences in roost-switching with a Kruskal–Wallis test, and for differences in roost-switching rates between the sexes with Wilcoxon rank-sum tests.

## 3. Results

**Figure 3.**
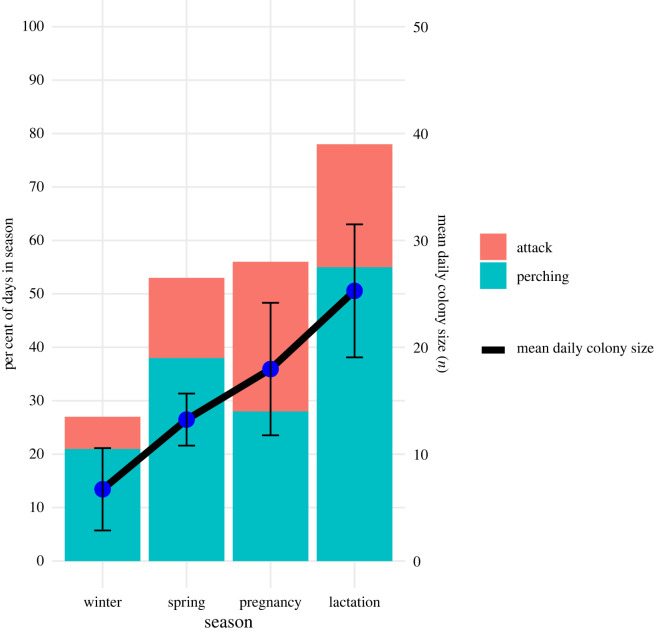
*Strix aluco* perching and hunting at the diurnal roosts of *N. lasiopterus* bats during four seasons. The height of the bars represents the percentage of all days per season during which *S. aluco* was observed perching and hunting at roosts. The green part of the bars represents the percentage of all days per season in which *S. aluco* was observed perching at the roost (owl behavioural event classes 2 and 3), the red part represents the percentage of all days per season in which *S. aluco* was observed attacking bats or roosts (event classes 4–6). For each day, only the highest event class was considered, so bars cannot exceed 100%. The blue dots represent the mean daily colony size of *N. lasiopterus* (sum of individuals in all roosts) per season, the error bars are the standard deviation. Number of observation days per season: winter = 70, spring = 34, pregnancy = 29, lactation = 64.

**Figure 4.**
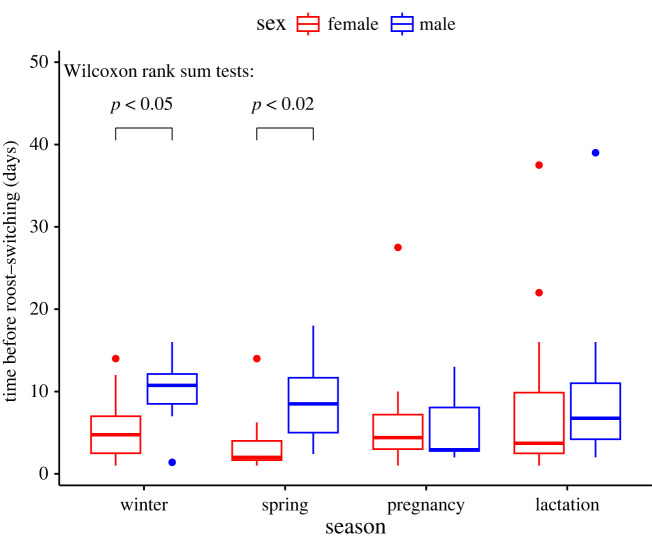
Roost-switching (days before switching) per season and sex in *N. lasiopterus* in the Doñana Biological Reserve, Andalusia, Spain. The lower and upper limits of the box represent the lower and upper quartiles (Q1, Q3), the ends of the whiskers are the lower and upper limits of the data, the dots are outliers, and the line in the boxes represents the median. The *p*-values of Wilcoxon rank sum tests are shown above the brackets for those seasons with a significantly different switching rate between the sexes.

The roosts were occupied by *N. lasiopterus* on average for 124 days (of 197 observation days) by a mean of 5.7 bats per roost (range 1–25 individuals, including bats with and without transponders). During our study period between January and July, we recorded 51 individually marked *N. lasiopterus* (34 females and 17 males) for on average 43 days (range 1–150 days). The number of individuals with transponders that were recorded at least once increased from 20 in winter (15 of which stayed in the colony for over 10 days during this season) to 37 in the lactation season (31 of which stayed for over 10 days during this season) (figure 3). The mean proportion of tagged bats per day in the study colony was 80%. The bats switched roosts every 6.0 ± 4.0 days (range of means: 1–15.8 days, figure 4) without any difference between seasons (Kruskal–Wallis test: *H* = 1.1, d.f. = 3, *p*-value = 0.8), but the roost-switching rate differed between the sexes over the full study period (Wilcoxon test: *W* = 144.5, *p* < 0.05, mean number of days before switching: females = 5.0 days, males = 7.8 days), and specifically in winter (Wilcoxon test: *W* = 22, *p* < 0.05) and spring (Wilcoxon test: *W* = 14, *p* < 0.02)

We observed three owl species, *Strix aluco*, *Athene noctua* and *Tyto alba*, at the bat roosts. *S. aluco* was recorded either perching or hunting (event categories 2–6) in front of at least one of the study roosts on 103 days, during which we observed in total 222 perching or hunting events (see data repository, exemplary videos of categories 1 to 6). On 37 days, we recorded four attacks by *S. aluco* directed at a roost (event category 4, data repository, video 4) and 38 attempted attacks on individuals of *N. lasiopterus* (categories 5–6, data repository, videos 5.1 to 5.5) of which at least three were successful captures (data repository, videos 6.1, 6.2). During diurnal inspections underneath roosts, we found two recently dead *N. lasiopterus* with claw wounds on their backs that probably resulted from an owl attack. Therefore, at least five *N. lasiopterus* were killed by owls during this study. Based on our observations and the territorial behaviour of *S. aluco* [39], we assumed one breeding pair of *S. aluco* at our study site.

We observed only nine capture attempts by *S. aluco* directed at pipistrelle bats, none of which was successful (data repository, video 7). *Athene noctua* was recorded only on 13 nights during which we observed 25 capture attempts that were all directed at pipistrelles, 17 of which were successful (data repository, videos 8.1, 8.2). *Tyto alba* was only observed perching at a roost on two occasions and there were no records of attacks.

**Figure 5.**
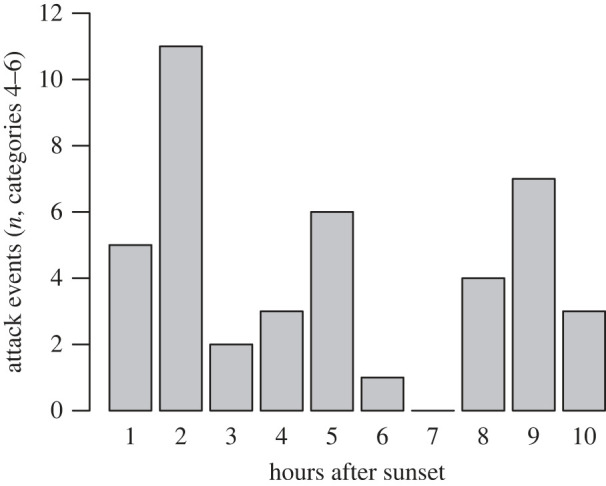
Number of attacks (event categories 4–6) of *S. aluco* on *N. lasiopterus* at roosts during the first 10 hours after sunset (*n* = 42 events).

We observed *S. aluco* perching and hunting at bat roosts during all seasons; however, days with owl hunting observations at roosts were least frequent during the winter season and owl hunting was most frequent during the lactation season (figure 3). Predation events by *S. aluco* occurred at all times of the night with a peak at around 2 h after sunset (figure 5, 42 events of categories 4–6). Most often the owls chased bats on the wing (86% of 42 attack events) often starting from a perch on or near to the bat roost from which the owl was able to track passing and approaching bats and those that tried to enter the roosts. Then the owls would either attempt to grab passing bats or chase flying bats. On just one occasion we recorded an attack of *S. aluco* directed at a bat emerging from the roost. This was not during the bat's first evening emergence at dusk, but later at night when the bat exited the roost after its first return (data repository, video 5.5). In a second type of attack event (12% of all attack events), the owls approached the roost entrance, and in some cases reached with their talons into the roost, most likely in an effort to tear a bat from the roost. When owls approached the roost entrance, there were usually bats inside the roost. In total, during 74% of all predation events (categories 4–6) at least 3 ± 3 *N. lasiopterus* were present inside the bat roost, based on PIT tag readings.

The emergence of *N. lasiopterus* from roosts was always straightforward, fast and without delay (typically less than 3 s, C Ibáñez & J Nogueras 2015, personal observation), and after emergence the bats gained altitude quickly in a direct flight. When returning to the roost, the bats often first approached the roost entrance without entering directly, and only entered after several approaches and landing attempts. Analysing PIT tag readings together with 98 video-recorded returns of bats to their roosts, we found that it may take up to 12 min from the first approach of a bat until it eventually enters the roost (17.5 ± 17.4 approaches and landing attempts, data repository, video 9.2).

**Figure 6.**
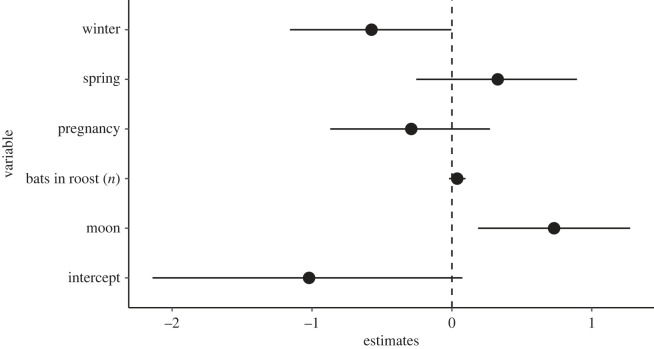
Parameter estimates of a GLMM on the effect of moon phase, number of bats in roosts and season on the incidence of *S. aluco* at roost of *N. lasiopterus*. Bars are 95% credible intervals. The reference category for season is ‘lactation’.

We found a positive effect of the moon phase on the presence of *S. aluco* at bat roosts, i.e. owls were more likely to perch at roosts at around full moon. However, we did not find any effect of season or the number of bats in a roost on the presence of *S. aluco* at roosts (figure 6, electronic supplementary material, table S1).

**Figure 7.**
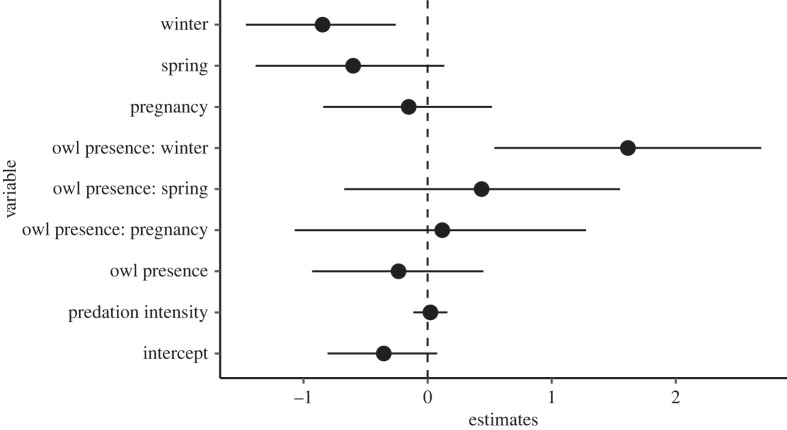
Parameter estimates of a GLMM on factors influencing roost-switching in *N. lasiopterus* roosting groups (less or equal/more roosting bats than the previous day). The analysis considers only data on *S. aluco* and *N. lasiopterus* in the dataset, all observation days are included (model ‘group size b’). Bars are 95% credible intervals. The reference category for season is ‘lactation’.

We did not find any effect of predation or of the presence of *S. aluco* on the roost-switching behaviour in *N. lasiopterus* in the models that tested for the number of bats in a roost compared with the previous day (models at the roost level). However, the models indicated that *N. lasiopterus* switched roosts less often in winter compared with the other seasons and that the presence of *S. aluco* reduced this effect (figure 7, see electronic supplementary material, figures S2 and S3 for additional model plots ‘group size a’ and ‘group size c’ and electronic supplementary material, table S2 for detailed results).

**Figure 8.**
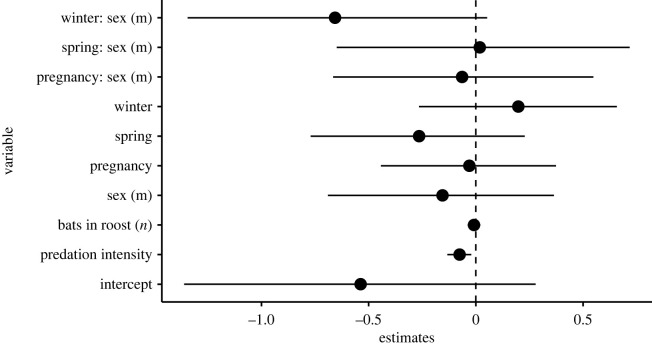
Parameter estimates of a GLMM on factors influencing the abandonment of a roost by individual *N. lasiopterus*. The analysis included data on *S. aluco* and *N. lasiopterus* only (model ‘gone b’), all observation days are included. Bars are 95% credible intervals. The reference category for season is ‘lactation’ and for sex ‘female’.

**Figure 9.**
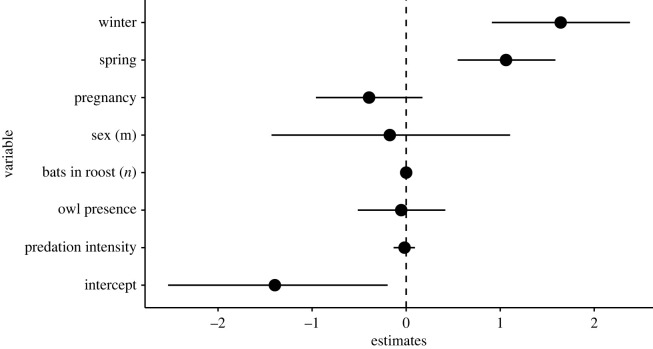
Parameter estimates of a GLMM on factors influencing the timing of emergence from roosts of individual *N. lasiopterus*. The analysis considered only the presence and predation by *S. aluco*. Bars are 95% credible intervals. The reference category for season is ‘lactation’ and for sex ‘female’.

The models that tested for effects at the level of the individual bat (models ‘gone a–c’) did not show any seasonal effects or effects of the bats' sex or the number of bats in a roost on the probability of roost-switching, while there was a slight, negative effect of *S. aluco* predation intensity on roost-switching in *N. lasiopterus*, i.e. bats switched roosts less often at higher predation intensities (figure 8, electronic supplementary material, table S3). Overall the results were similar for all models whether they were only testing for interactions between *S. aluco* and *N. lasiopterus* or owl predation in general, i.e. whether the days with predation events of *A. noctua* or the incidence of events of other owl species were included in the dataset or not (electronic supplementary material, figures S4 and S5 for models ‘gone a’ and ‘gone c’). We did not find any effect of owl presence or predation, the bats' sex or the number of bats in the roost on the time of emergence of individual *N. lasiopterus*, but there was a positive effect on a later emergence in winter and spring (figure 9, electronic supplementary material, table S4).

## 4. Discussion

We regularly observed predation behaviour of tawny owls (*Strix aluco*) at the diurnal roosts of a small breeding colony of greater noctule bats (*Nyctalus lasiopterus*) in southern Spain. The owls' predation behaviour was clearly focused on the bats, because the birds perched at bat roosts, and actively watched and hunted approaching bats. We only once recorded an owl with a captured small rodent. Despite the risk of owl predation close to bat roosts on most days during our study, the data do not support the hypothesis that owl predation triggers roost-switching as an anti-predation behaviour in *N. lasiopterus*, or that owl predation events thereby directly influence the fission–fusion behaviour in this species. Popa-Lisseanu *et al*. [26], studying fission–fusion in a *N. lasiopterus* population in Seville, Spain, also discarded predation as a principal factor triggering roost-switching, due to the bats' continued use of the same roosts (although roosting group composition changed frequently). While other studies have found parasite infestation of roosts, the roosts' thermal conditions or the weather to influence roost-switching in other bat species [22,23,40], these factors seem unlikely to explain roost-switching in our study system, because we did not observe roost abandonment by entire roosting groups for longer time periods or simultaneous roost-switching that could disrupt parasite cycles. We cannot, however, rule out individual thermal strategies as a driver of the observed individual roost-switching. Other explanations for roost-switching within a fission–fusion context relate to group stability and to information transfer within the social group [41,42]. Popa-Lisseanu *et al*. [26] assumed for *N. lasiopterus* that roost-switching serves to maintain within-group social bonds and transfer of information on roost availability. Interestingly, we found a mean time before roost-switching of 6.0 days, with males staying for significantly longer time periods in roosts than females. This is more than double the mean roosting period of 2.7 days that was reported by Popa-Lisseanu *et al*. [26] for the Seville population, and it is also a much lower roost-switching rate than for other closely related species (e.g. 2.6 days in *N. leisleri* [43]). This relatively low average roost-switching rate may be related to the smaller size of the study colony, the much lower local roost availability, and possibly to the higher proportion of males in the Doñana colony than in the Seville population [26]. In the Seville population, over 200 individuals from three social groups used over 70 roosts, which may require more information on roost utilization and a much more dynamic roost selection to maintain social bonds and colony coherence compared with the single social group dispersed over only 12 roosts, most within an area of *ca* 2 hectares, studied here [26]. A previous telemetry study also found that *N. lasiopterus* in the Doñana Reserve only roosts in the artificial bat boxes due to a lack of natural roosts [31]. The higher proportion of males in our study group and their higher roost fidelity may be related to the low roost availability that impedes a stricter sexual segregation. The weak negative effect of owl predation on roost-switching indicated by the model for individual roost-switching (figure 8, model ‘gone b’) is somewhat surprising. Given the large dataset, the models at the roost group level in particular appear to be robust. However, the small number of successful owl predation events during a large observation period may have resulted in a small number of PIT-tagged individuals affected by owl predation, which may still allow for some bias in the data that cannot be adequately explained by the model variables, or most of the posterior distribution of the effect might not be beyond a meaningful threshold (see electronic supplementary material, table S3).

There is little information on the ability of bats to detect predators either visually or acoustically, when they are not being directly attacked [14]. However, we believe that the unclear effects of the threat of predation on roost-switching cannot be entirely explained by an inability of the bats to notice predation risks. In the majority of events bats were present in the roosts, and the owls hunted very close to the roost entrance, often perching on the roost itself. Also, during some predation events, the owls tried to pull bats from the roost by reaching their talons into the roost. Although our study did not include acoustic recordings, we assume that attacked bats may have uttered distress calls that would probably have been noticed by other bats inside the roost. Distress calls are common in captured *N. lasiopterus* (C Ibáñez & J Nogueras 2015, personal observation), and they are uttered by many bat species under physical stress, e.g. when being seized by a predator. Distress calls have been found to attract other bats, and presumably warn kin, distract predators and induce mobbing [44–48]. Therefore, we assume that bats in the observed roosts were capable of noticing predation events. Considering the general sensitivity of bats to roost disturbances, the roost fidelity observed here is somewhat remarkable. Other studies have shown that intensive owl predation or activity near bat roosts can result in at least partial or temporary roost abandonment, but interspecific comparisons are complicated by species' different roosting and social behaviours (e.g. cave versus tree roosting; a lack of fission–fusion behaviour [15,16]). Here, even if the bats did perceive the predation risk, they may have gauged the risk to be insufficient to require a behavioural response, or perhaps other constraints prevented the bats from switching roosts. For our study colony, we suspect that limited roost availability contributed to the bats' relatively high roost fidelity, because the colony used only 12 roosts in a small patch of trees in a large grassland flood plain and scrubland that is unlikely to provide a large quantity of alternative nearby roosts [31]. Furthermore, in the study area, a larger colony of *Pipistrellus pygmaeus* inhabits similar artificial roosts, often with more than 50 individuals in a roost simultaneously, but we never observed *N. lasiopterus* roosting with pipistrelles. Therefore, roost competition may have limited the bats' options for roost-switching. Contrary to this assumption, however, some study roosts were unoccupied at all times, meaning that there was the potential for roost-switching within the area. In this respect, we disregard the thermal properties of roosts or low roost temperatures to significantly influence roost occupation, as the local climate during the study period was mostly temperate/warm. Moreover, roost-switching to escape predation may be an inefficient strategy in the studied colony, since a few roosts within a small forest patch can be patrolled rapidly by an acoustically oriented predator, due to the frequent, loud vocalizations of *N. lasiopterus* that are also audible to the human ear (C Ibáñez & J Nogueras 2015, personal observation). We acknowledge that the increase in disturbance at roosts across the classes of owl behaviour at roosts that we have used to define the variable ‘predation intensity’ may not be linear, as we cannot know the gradual increase in disturbance perceived by bats from one event class to the next, and predation may be perceived differently between individuals and situations. However, we assume that the categorization improves the precision of the models compared with a simple presence/absence model that neglects the frequency of owl presence and repeated hunting events per night.

If roost abandonment is influenced by group decision-making, it is possible that the number of group members that experienced a predation event was often insufficient to initiate roost-switching. In *Myotis bechsteinii*, another species with a fission–fusion society, field experiments have shown that the abandonment of a roost after disturbance can be influenced by majority group decisions [49]. Moreover, conflicting individual decisions, which may depend on individuals' physiological status, age or personality [50], may influence group behaviour and fission–fusion movements [51]. It is possible that beyond a certain cost associated with roost-switching, bats may prefer to remain in their present roost despite the risk of predation.

Some authors have suggested that a perceived predation risk may influence the timing of bats’ emergence from their roost [52,53], and some studies have found that the distress calls of captured bats near the roost delay the emergence of bats from within roosts [52,54,55]. However, we did not find an effect of the threat of owl predation on the bats' emergence time. This is perhaps not surprising given that the majority of owl hunting and predation happened later at night, well beyond sunset and the bats’ emergence from roosts. Additionally, the bats' fast, direct flight away from the roost into open space, and rapid height gain after emergence, may complicate predation for perch hunters.

We acknowledge that the identity of the attacked bats and their individual associations with the observed roosts remain unknown from video recordings. Also, due to the video cameras’ restricted field of view some owl occurrences and predation events might not have been recorded. Moreover, our categorization of owl behaviour might not sufficiently reflect how the bats perceive owl presence and predation, and this may have contributed to the lack of an effect of predation on bat behaviour in our models. For example, we defined as an attack on the roost only those owl movements that resulted in direct contact with the roost. However, in some cases the owls hovered in front of the roost without making any contact, and these events were regarded as passing flights (data repository, video 1.2). It is possible that bats inside the roosts noticed these approaching predators and expressed a behavioural response to their presence.

The model indicated a higher probability of owl presence at roosts closer to the full moon. We acknowledge that cloud cover may have an obscuring effect on the factor ‘moon phase’. However, as the probability of clouds is evenly distributed over a large part of the study period, we assume that cloud cover may weaken the strength of the result, but does not lead to a systematic bias.

The owls’ hunting strategy is remarkable, as they mostly prey on bats on the wing, after dusk during poor light, and also during the bats' return to the roost, a time at which the bats’ occurrence is less predictable than during emergence. It is unclear whether the owls used visual or acoustic cues to detect bats. Indeed, this hunting strategy is not well documented in the literature, possibly because it is difficult to observe. Previous reports of owls hunting bats at roosts differ significantly from the owl predation observed in this study. While other authors have described the short-term, intense effects of seemingly exceptional predation events [15,16], we observed a continuous predation risk on most days over our seven-month study period. This low-intensity predation by *S. aluco* appears to have continued over several years at least, judging from video recordings from before and after the study period (C Ibáñez & J Nogueras 2018, personal communication). This contrasts with other observations of predation by *S. aluco* at bat roosts, e.g. on *M. emarginatus*, where, during three breeding periods, predation was found to concentrate exclusively on a few weeks during 1 year [16].

We observed that *N. lasiopterus* often makes several roost approaches and landing attempts before entering the roost, a behaviour that has been observed in other bat species also [15,27,56], and that has been referred to as ‘dawn swarming’ [57,58,59]. However, the observed behaviour does not truly conform with ‘swarming’, because bats mostly arrived at and entered the roosts during the night and one after the other rather than flying in groups around roosts. The function of swarming in the literature is commonly associated with locating of roosts, group cohesion and recruitment, and it has been assumed that it may increase predation risk [57]. We argue that the repeated roost approaches that we observed in *N. lasiopterus* allow inspection of the roost and assessment of the current predation risk by locating predators and triggering attacks that are easy to avoid. Once the bats land at the roost entrance before climbing into the roost, they are very exposed and vulnerable to predation. Indeed, we observed various unsuccessful predation attempts by an owl chasing a bat after it turned away from the roost entrance. Owl attacks on the roosts themselves (event class 4) occurred mostly later at night with bats inside the roosts and did not seem to be triggered by approaching bats. Therefore, it is possible that the owls were attracted by noise from the bats inside the roost (data repository, videos 1.2 and 4).

Despite the frequent presence of *S. aluco* at bat roosts, we assume that the proportion of bats in the owls' diet is very low. Previous studies have found the mean proportion of bats in the diet of *S. aluco* across Europe to be less than 1% [9]. The daily food intake of *S. aluco* has been estimated to be between 53 g [60] and 180 g [13], corresponding to 2–7 small rodents (e.g. field voles). Nestling food uptake has been estimated to be *ca* 57–88 g per day [61,62]. Although it is unclear whether both individuals of the resident owl pair preyed on bats, we did record two owls at roosts simultaneously (data repository, video 5.4). Consequently, with the average number of three nestlings for an average 32 days before fledging, the total food requirements of the breeding owl pair and its offspring would be at least 60.8 kg during the study period, taking into account that the fledged offspring may remain in the parents' territory for two–three months after fledging [61]. Based on these approximate calculations, the three successful attacks on *N. lasiopterus*, with an average body mass of 48 g [28], may have contributed less than 0.3% of the owls’ overall food intake during the study period. Even if the video recordings did not capture all owl predation, the proportion of bats in the diet of *S. aluco* in Doñana is probably similar to estimates from other studies in Europe [63], and far below the maximum 30% recorded contribution of bats to the diet of *S. aluco* that temporarily specialized on bats [9]. Considering that we recorded also only nine capture attempts on pipistrelles, none of which was successful, we conclude that the local breeding pair of *S. aluco* only opportunistically preyed on bats [64]. Unfortunately, we were not able to analyse owl pellets, a widely used method for studying owl diets, because pellet collection was complicated by a very high abundance of boar and other scavengers of owl pellets (C Ibáñez & J Nogueras 2015, personal observation).

We recorded *A. noctua* hunting pipistrelle bats, but never *N. lasiopterus.* Although *A. noctua* is known to prey on animals of at least the size of *N. lasiopterus* (e.g. *Rattus rattus* [65]), hunting the large and fast flying *N. lasiopterus* might require a much higher effort than focusing on the more abundant pipistrelle bats.

From the perspective of *N. lasiopterus*, owl predation had a significant impact on the local population. Besides the three *N. lasiopterus* that we recorded to have fallen prey to *S. aluco*, we found two dead individuals under roosts with claw wounds that were probably caused by an avian predator. As it is unclear how many bats fell prey to *S. aluco* away from roosts, the recorded number of bat casualties is a minimum value. Assuming the colony size to comprise 51 to 64 bats (estimated from the number of PIT-tagged bats and their proportion with unmarked bats), five individuals predated by *S. aluco* would represent 8–10% of the colony during seven months of observations that include the period of pregnancy and lactation with the highest number of bats in the colony. For the second half of the year, we assume that fewer bats fall prey to *S. aluco*, because from August to January the number of bats in the colony usually declines, as most bats move to their wintering sites. Furthermore, the resident pair of *S. aluco* would have a lower food demand outside their breeding season. Papadatou *et al*. [66] estimated for the nearby Seville colony of *N. lasiopterus* a survival rate of 0.74. Assuming a similar survival rate in the study colony, *S. aluco* predation alone would account for around 30–40% of the colony's mortality and is therefore an important factor for colony stability.

## 5. Conclusion

We did not find any clear indication that owl predation triggers roost-switching or delayed emergence from roosts as an anti-predator behaviour in *N. lasiopterus.* It remains unclear whether the bats were unable to detect predators and perceive the predation risk, or whether other factors, such as low roost availability obliged the bats to remain in their roosts despite the predation risk. Although our observations confirm previous findings that bats are of minor importance in the diet of owls, we show a major impact of owl predation on the study colony of *N. lasiopterus*. This is a very rare bat species (Vulnerable in the IUCN red list [67]) that depends on cavity-rich old-growth forests for roosting [26,68]. In heavily deforested southern Spain, the species has also been found occasionally roosting in exotic tree species in an urban environment, such as a city park in Seville or the Zoo in Jerez de la Frontera [26,31], and the population is fragmented, with some very localized roosting sites, some of which are threatened by deforestation, unsuitable forestry practices, wind energy development and aggressive competition for roosts with exotic parakeets (*Psittacula krameri*) [67,69,70,71].

Our study therefore highlights the significant impact that even low-intensity owl predation can have particularly on small, local bat populations, which is of particular importance for the conservation of bat species. We propose that a low roost availability may prevent bats from switching roosts to escape predation, and make their movements more predictable, which in turn may increase the predators' predation success. In this regard, our study shows that even typical natural levels of owl predation can significantly impact bat survival, and decisively endanger the stability of small and isolated bat colonies.

## Data Availability

The data and videos supporting the results of this study, and the R code are available from the OSF repository: https://doi.org/10.17605/OSF.IO/WDMSZ [72]. We included further graphs supporting our results in the electronic supplementary material [73].

## References

[1] Rydell J, Speakman JR. 1995 Evolution of nocturnality in bats: potential competitors and predators during their early history. Biol. J. Linn. Soc. **54**, 183-191. (10.1111/j.1095-8312.1995.tb01031.x)

[2] Boinski S, Timm RM. 1985 Predation by squirrel monkeys and double-toothed kites on tent-making bats. Am. J. Primatol. **9**, 121-127. (10.1002/ajp.1350090205) 32102491

[3] Sparks DW, Roberts KJ, Jones C. 2000 Vertebrate predators on bats in North America north of Mexico. Reflections of a naturalist: papers honoring Professor Eugene D. Fleharty. Fort Hays Studies. Special issue **1**, 229-241.

[4] Esbérard CEL, Vrcibradic D. 2007 Snakes preying on bats: new records from Brazil and a review of recorded cases in the Neotropical region. Rev. Bras. Zool. **24**, 848-853. (10.1590/S0101-81752007000300036)

[5] Goodman SM, Razakaratrimo SVJ, de Roland LAR. 2016 An analysis of bat hawk *Macheiramphus alcinus* diet in the Melaky region of lowland western Madagascar. Ostrich **87**, 77-80. (10.2989/00306525.2015.1104395)

[6] Seijas AE. 1996 Feeding of the bat falcon (*Falco rufigularis*) in an urban environment. J. Raptor Res. **30**, 33-35.

[7] Ibáñez C, Ramo C, Busto B. 1992 Notes on food habits of the black and white owl. Condor, **94**, 529-531. (10.2307/1369226)

[8] Gerhardt RP, Gerhardt DM, Flatten CJ, González NB. 1994 The food habits of sympatric *Ciccaba* owls in Northern Guatemala. J. Field Ornithol. **65**, 258-264.

[9] Sieradzki A, Mikkola H. 2020 A review of European owls as predators of bats. In Owls (ed. H Mikkola), pp. 1-20. London: IntechOpen.

[10] Sommer RS, Niederle M, Labes R, Zoller H. 2009 Bat predation by the barn owl *Tyto alba* in a hibernation site of bats. Folia Zool. **58**, 98-103.

[11] Tian L, Zhou X, Shi Y, Guo Y, Bao W. 2015 Bats as the main prey of wintering long-eared owl (*Asio otus*) in Beijing: integrating biodiversity protection and urban management. Integr. Zool. **10**, 216-226. (10.1111/1749-4877.12123) 25316405

[12] Barclay RMR, Harder LD. 2003 Life histories of bats: life in the slow lane. In Bat ecology (eds TH Kunz, MB Fenton), pp. 209-253. Chicago, IL: University of Chicago Press.

[13] Speakman, JR. 1991 The impact of predation by birds on bat populations in the British Isles. Mamm. Rev. **21**, 123-142. (10.1111/j.1365-2907.1991.tb00114.x)

[14] Lima SL, O'Keefe JM. 2013 Do predators influence the behaviour of bats? Biol. Rev. **88**, 626-644. (10.1111/brv.12021) 23347323

[15] Barclay RMR, Thomson CE, Phelan FJS. 1982 Screech owl, *Otus asio*, attempting to capture little brown bats, *Myotis lucifugus*, at a colony. Can. Field Nat. **96**, 205-206.

[16] Spitzenberger F, Engelberger S, Kugelschafter K. 2014 Real time observations of *Strix aluco* preying upon a maternity colony of *Myotis emarginatus*. Vespertilio **17**, 185-196.

[17] Speakman JR, Stone RE, Kerslake JE. 1995 Temporal patterns in the emergence behaviour of pipistrelle bats, *Pipistrellus pipistrellus*, from maternity colonies are consistent with an anti-predator response. Anim. Behav. **50**, 1147-1156. (10.1016/0003-3472(95)80030-1)

[18] Petrželková KJ, Zukal J. 2003 Does a live barn owl (*Tyto alba*) affect emergence behavior of serotine bats (*Eptesicus serotinus*)? Acta Chiropt. **5**, 177-184. (10.3161/001.005.0202)

[19] Kalcounis MC, Brigham RM. 1994 Impact of predation risk on emergence by little brown bats, *Myotis lucifugus* (Chiroptera: Vespertilionidae), from a maternity colony. Ethology **98**, 201-209. (10.1111/j.1439-0310.1994.tb01071.x)

[20] Lewis SE. 1995 Roost fidelity of bats: a review. J. Mammal. **76**, 481-496. (10.2307/1382357)

[21] Patriquin KJ, Ratcliffe JM. 2016 Should I stay or should I go? Fission–fusion dynamics in bats. In Sociality in bats (ed. J Ortega), pp. 65-103. Cham, Switzerland: Springer.

[22] Kerth G, Weissmann K, König B. 2001 Day roost selection in female Bechstein's bats (*Myotis bechsteinii*): a field experiment to determine the influence of roost temperature. Oecologia **126**, 1-9. (10.1007/s004420000489) 28547427

[23] Reckardt K, Kerth G. 2007 Roost selection and roost-switching of female Bechstein's bats (*Myotis bechsteinii*) as a strategy of parasite avoidance. Oecologia **154**, 581-588. (10.1007/s00442-007-0843-7) 17805579

[24] Willis CK, Brigham RM. 2004 Roost switching, roost sharing and social cohesion: forest-dwelling big brown bats, *Eptesicus fuscus*, conform to the fission–fusion model. Anim. Behav. **68**, 495-505. (10.1016/j.anbehav.2003.08.028)

[25] O'Donnell CFJ, Sedgeley JA. 2006 Causes and consequences of tree-cavity roosting in a temperate bat, *Chalinolobus tuberculatus*, from New Zealand. In Functional and evolutionary ecology of bats (eds A Zubaid, GF McCracken, TH Kunz), pp. 308-321. New York, NY: Oxford University Press.

[26] Popa-Lisseanu AG, Bontadina F, Mora O, Ibáñez C. 2008 Highly structured fission–fusion societies in an aerial-hawking, carnivorous bat. Anim. Behav. **75**, 471-482. (10.1016/j.anbehav.2007.05.011)

[27] Russo D, Cistrone L, Jones G. 2005 Spatial and temporal patterns of roost use by tree-dwelling barbastelle bats *Barbastella barbastellus*. Ecography **28**, 769-776. (10.1111/j.2005.0906-7590.04343.x)

[28] Ibáñez C, Juste J, García-Mudarra JL, Agirre-Mendi PT. 2001 Bat predation on nocturnally migrating birds. Proc. Natl Acad. Sci. USA **98**, 9700-9702. (10.1073/pnas.171140598) 11493689 PMC55515

[29] Ibáñez C, Popa-Lisseanu AG, Pastor-Beviá D, García-Mudarra JL, Juste J. 2016 Concealed by darkness: interactions between predatory bats and nocturnally migrating songbirds illuminated by DNA sequencing. Mol. Ecol. **25**, 5254-5263. (10.1111/mec.13831) 27575398

[30] Santos JD, Meyer CFJ, Ibáñez C, Popa-Lisseanu AG, Juste J. 2016 Dispersal and group formation dynamics in a rare and endangered temperate forest bat (*Nyctalus lasiopterus*, Chiroptera: Vespertilionidae). Ecol. Evol. **6**, 8193-8204. (10.1002/ece3.2330) 27878088 PMC5108270

[31] Popa-Lisseanu AG, Bontadina F, Ibáñez C. 2009 Giant noctule bats face conflicting constraints between roosting and foraging in a fragmented and heterogeneous landscape. J. Zool. **278**, 126-133. (10.1111/j.1469-7998.2009.00556.x)

[32] Henry M, Thomas DW, Vaudry R, Carrier M. 2002 Foraging distances and home range of pregnant and lactating little brown bats (*Myotis lucifugus*). J. Mamm. **83**, 767-774. (10.1644/1545-1542(2002)083<0767:FDAHRO>2.0.CO;2)

[33] Kurta A, Murray SW, Miller DH. 2002 Roost selection and movements across the summer landscape. In The Indiana bat: biology and management of an endangered species (eds A Kurta, J Kennedy), pp. 118-129. Austin, TX: Bat Conservation International.

[34] Kunz TH, Hood WR. 2000 Parental care and postnatal growth in the Chiroptera. In Reproductive biology of bats (eds EG Crichton, PH Krutzsch), pp. 415-468. San Diego, CA: Academic Press.

[35] Hernández-Mijangos LA, Horváth A, Canales RP. 2009 Observations on female bats transporting non-volant juveniles during flight. Chiropt. Neotrop. **15**, 472-476.

[36] Bürkner PC. 2017 brms: An R package for Bayesian multilevel models using Stan. J. Stat. Softw. **80**, 1-28. (10.18637/jss.v080.i01)

[37] R Core Team. 2021 *R: A language and environment for statistical computing*. Vienna, Austria: R Foundation for Statistical Computing. See https://www.R-project.org/.

[38] Hartig F. 2022 DHARMa: residual diagnostics for hierarchical (multi-level/mixed) regression models. R package version 0.4.5.

[39] Sunde P, Bølstad MS. 2004 A telemetry study of the social organization of a tawny owl (*Strix aluco*) population. J. Zool. **263**, 65-76. (10.1017/S0952836904004881)

[40] Patriquin KJ, Leonard ML, Broders HG, Ford WM, Britzke ER, Silvis A. 2016 Weather as a proximate explanation for fission–fusion dynamics in female northern long-eared bats. Anim. Behav. **122**, 47-57. (10.1016/j.anbehav.2016.09.022)

[41] Kerth G, Reckardt K. 2003 Information transfer about roosts in female Bechstein's bats: an experimental field study. Proc. R. Soc. B **270**, 511-515. (10.1098/rspb.2002.2267) PMC1691266 12641906

[42] Ratcliffe JM, Ter Hofstede HM. 2005 Roosts as information centres: social learning of food preferences in bats. Biol. Lett. **1**, 72-74. (10.1098/rsbl.2004.0252) 17148131 PMC1965196

[43] Spada M et al. 2008 Roost selection by non-breeding Leisler's bats (*Nyctalus leisleri*) in montane woodlands: implications for habitat management. Acta Chiropt. **10**, 81-88. (10.3161/150811008X331117)

[44] Russ JM, Racey PA, Jones G. 1998 Intraspecific responses to distress calls of the pipistrelle bat, *Pipistrellus pipistrellus*. Anim. Behav. **55**, 705-713. (10.1006/anbe.1997.0665) 9514675

[45] Lučan RK, Šálek M. 2013 Observation of successful mobbing of an insectivorous bat, *Taphozous nudiventris* (Emballonuridae), on an avian predator, *Tyto alba* (Tytonidae). Mammalia **77**, 235-236. (10.1515/mammalia-2012-0067)

[46] Knörnschild M, Tschapka M. 2012 Predator mobbing behaviour in the greater spear-nosed bat, *Phyllostomus hastatus*. Chiroptera Neotropical **18**, 1132-1135.

[47] Carter G, Schoeppler D, Manthey M, Knörnschild M, Denzinger A. 2015 Distress calls of a fast-flying bat (*Molossus molossus*) provoke inspection flights but not cooperative mobbing. PLoS ONE **10**, e0136146. (10.1371/journal.pone.0136146) 26353118 PMC4564210

[48] Chaverri G, Ancillotto L, Russo D. 2018 Social communication in bats. Biol. Rev. **93**, 1938-1954. (10.1111/brv.12427) 29766650

[49] Kerth G, Ebert C, Schmidtke C. 2006 Group decision making in fission–fusion societies: evidence from two-field experiments in Bechstein's bats. Proc. R. Soc. B **273**, 2785-2790. (10.1098/rspb.2006.3647) PMC1635504 17015328

[50] Menzies AK, Timonin ME, McGuire LP, Willis CKR. 2013 Personality variation in little brown bats. PLoS ONE **8**, e80230. (10.1371/journal.pone.0080230) 24312205 PMC3842255

[51] Fleischmann D et al. 2013 Female Bechstein's bats adjust their group decisions about communal roosts to the level of conflict of interests. Curr. Biol. **23**, 1658-1662. (10.1016/j.cub.2013.06.059) 23954425

[52] Fenton MB, Rautenbach IL, Smith SE, Swanepoel CM, Grosell J, Van Jaarsveld J. 1994 Raptors and bats: threats and opportunities. Anim. Behav. **48**, 9-18. (10.1006/anbe.1994.1207)

[53] Duvergé PL, Jones G, Rydell J, Ransome RD. 2000 Functional significance of emergence timing in bats. Ecography **23**, 32-40. (10.1111/j.1600-0587.2000.tb00258.x)

[54] McWilliam AN. 1989 Emergence behaviour of the bat *Tadarida* (*Chaerephon*) *pumila* (Chiroptera: Molossidae) in Ghana, West Africa. J. Zool. **219**, 698-701. (10.1111/j.1469-7998.1989.tb02615.x)

[55] Wu X, Pang Y, Luo B, Wang M, Feng J. 2018 Function of distress calls in least horseshoe bats: a field study using playback experiments. Acta Chiropt. **20**, 455-464. (10.3161/15081109ACC2018.20.2.015)

[56] Lewis SE. 1996 Low roost-site fidelity in pallid bats: associated factors and effect on group stability. Behav. Ecol. Sociobiol. **39**, 335-344. (10.1007/s002650050298)

[57] Naďo L, Kaňuch P. 2013 Dawn swarming in tree-dwelling bats—an unexplored behaviour. Acta Chiropt. **15**, 387-392. (10.3161/150811013X679008)

[58] Naďo L, Kaňuch P. 2015 Swarming behaviour associated with group cohesion in tree-dwelling bats. Behav. Processes **120**, 80-86. (10.1016/j.beproc.2015.09.005) 26367198

[59] Ružinská R, Lőbbová D, Kaňuch P. 2022 Demographic characteristics shape patterns of dawn swarming during roost switching in tree-dwelling Daubenton's bat*.* Sci. Rep. **12**, 1-10. (10.1038/s41598-022-14246-2) 35705697 PMC9200770

[60] Contoli L, Sammuri G. 1978 Predation on small mammals by tawny owl and comparison with barn owl in the Farma valley (central Italy). Ital. J. Zool. **45**, 323-335. (10.1080/11250007809440140)

[61] Cramp S. 1985 The birds of the Western Palearctic, vol. 4. Oxford, UK: Oxford University Press.

[62] Stave M. 2016 Prey deliveries at nests of the tawny owl (*Strix aluco*): diet and diel pattern. Master's thesis, Norwegian University of Life Sciences, Ås.

[63] Lesiński G, Gryz J, Kowalski M. 2009 Bat predation by tawny owls *Strix aluco* in differently human-transformed habitats. Ital. J. Zool. **76**, 415-421. (10.1080/11250000802589535)

[64] Lesiński G, Kasprzyk K, Gryz J. 2012 Bats taken by the tawny owl (*Strix aluco*) in relation to its roosting site. *North-West**.* J. Zool. **8**, 247-251.

[65] Gotta A, Pigozzi G. 1997 Trophic niche of the barn owl and little owl in a rice field habitat in northern Italy. *Ital**.* J. Zool. **64**, 55-59. (10.1080/11250009709356172)

[66] Papadatou E et al. 2012 Comparing survival among species with imperfect detection using multilevel analysis of mark–recapture data: a case study on bats. Ecography **35**, 153-161. (10.1111/j.1600-0587.2011.07084.x)

[67] Alcaldé J, Juste J, Paunović M. 2016 *Nyctalus lasiopterus*. *The IUCN Red List of Threatened Species* 2016: e.T14918A22015318.

[68] Russo D, Mäenurm A, Martinoli A, Zotti M, Cistrone L. 2023 Forest islands in farmland provide vital roost trees year-round for giant and common noctule bats: management implications. For. Ecol. Manag. **540**, 121053. (10.1016/j.foreco.2023.121053)

[69] Sánchez-Navarro S, Rydell J, Ibáñez C. 2019 Bat fatalities at wind-farms in the lowland Mediterranean of southern Spain. Acta Chiropt. **21**, 349-358. (10.3161/15081109ACC2019.21.2.010)

[70] Sánchez-Navarro S, Gálvez-Ruiz D, Rydell J, Ibáñez C. 2023 High bat fatality rates estimated at wind-farms in southern Spain. Acta Chiropt. **25**, 125-134. (10.3161/15081109ACC2023.25.1.007)

[71] Hernández-Brito D, Carrete M, Ibáñez C, Juste J, Tella JL. 2018 Nest-site competition and killing by invasive parakeets cause the decline of a threatened bat population. R. Soc. Open Sci. **5**, 172477. (10.1098/rsos.172477) 29892437 PMC5990744

[72] Kelm DH, Ibáñez C. 2023 Supporting information: Continuous intensity predation by owls (Strix aluco) on bats (Nyctalus lasiopterus) in Spain and the potential effect on bat colony stability. OSF (10.17605/OSF.IO/WDMSZ) PMC10427821 37593707

[73] Kelm DH, Langheld M, Nogueras J, Popa-Lisseanu AG, Ibáñez C. 2023 Continuous low-intensity predation by owls (*Strix aluco*) on bats (*Nyctalus lasiopterus*) in Spain and the potential effect on bat colony stability. Figshare. (10.6084/m9.figshare.c.6777784) PMC10427821 37593707

